# A New Solvent for Phosphorus

**Published:** 1874-01

**Authors:** A. W. Gerrard

**Affiliations:** Dispenser and Teacher of Pharmacy to the University College Hospital


### A New Solvent of Phosphorus; Its Preparation and Pharmaceutical Use.

By A. W. Gerrard, Dispenser and Teacher of Pharmacy to the University College Hospital.

The principal known and most generally adopted solvents of phosphorus in pharmaceutical purposes are bisulphide of carbon, chloroform, ether, alcohol, oil of almonds, oil of theobroma, and mutton suet.

The power of these bodies to dissolve this element varies from any proportion to less than half per cent.; the most powerful being bisulphide of carbon, the least, alcohol.

Most of the above solutions of phosphorus, when dispensed, are of an unsatisfactory and unstable character. Those which are fluid and miscible with water in the presence of mucilage—the manner in which it is usually prescribed—are rapidly decomposed and become inert; they are likewise nauseous and objectionable to the patient in an extreme degree. The solid forms are but little better and are exceedingly troublesome to manipulate.

Bisulphide of carbon has been recommended by Mr. Proctor, of Newcastle, as a means of dispensing phosphorus in the pill form, and it answers the purpose very well, with the exception that the pills retain a compound smell of phosphorus and bisulphide of carbon, which is repulsive in the utmost degree.

The new substance which I propose to add to the list of solvents of phosphorus is resin, that body described in the Pharmacopœia as the "residue of the distillation of the turpentine." This substance suggested itself to me amongst others as a probable solvent, and the result of my experiments upon it is that I

have found it capable of dissolving four or more per cent. of phosphorus; the limit of its solubility is a question for further experiment.

I would call this substance phosphoretted resin. The method of preparing it is thus: Take a strong wide-mouthed well-stoppered bottle and weigh it, then melt a quantity of resin sufficient to fill the bottle; let the bottle be warmed, then pour in the resin to nearly, but not quite fill the bottle, reweigh, and for every ninety-six parts of resin take four of phosphorus. Now observe that the resin is in a fluid state; if so, add the phosphorus, and fix the stopper tightly. Place in a sand-bath previously warmed, and apply heat to 200°C. or 392 F.; digest at this temperature, and shake frequently until the phosphorous is dissolved.

The kind of resin to be used in this preparation is the black translucent variety, known in commerce as rosin, not that pale, yellowish kind, usually met with in chemists' shops, unless it has previously been deprived of its water, of which it contains a varying amount, sometimes ten per cent.

In conducting the process, it is necessary to observe the following precautions: in adding the phosphorous, if possible let it be in one piece, and take care that the resin is previously in a fluid condition, as then the phosphorus readily sinks below the surface, and is covered by the resin; otherwise, if the phosphorus were in small pieces and the resin semi-fluid, the phosphorus would rest on the half-hot resin, and speedily take fire; but by observing the above precautions, this accident may be prevented.

A bottle full of the preparation should be made at a time, as I find there is great risk of accident (having had one myself) if the vessel is only partly filled. The phosphorous is also volatilized, and deposited in the upper portion of the bottle.

Keep a thermometer in the sand-bath during the process, and maintain the temperature between 200° and 210°C. At higher temperatures the resin boils, and the heat is liable to change the phosphorous to the red amorphous state.

When the prepared resin has cooled it is difficult to remove it unless the bottle be broken; the method I have adopted is to draw it from the bottle, when partly cooled, under hot water.

It is a pharmaceutical process which, like many others, requires care and attention to ensure success, but whatever difficulties may arise, to a practical person a remedy will suggest itself.

I will here mention a curious change which takes place if this phosphoretted resin be reheated. When it reaches a certain temperature it becomes of a whitish cream color throughout; if the temperature be raised still higher it again becomes transparent; this phenomenon does not occur in the cooling. It is probably due to the influence of molecular change.

The formula I would suggest for its exhibition is the following: Take of—

Phosphoretted Resin, 4 per cent ............. 25 grains.
Powdered White Sugar ....  ............... 75 grains.
Tincture of Tolu, a sufficient quantity.

Pulverize the resin, mix with the sugar, and form into a mass with tincture of tolu,—eight to ten drops are sufficient; then divide into twenty pills, each pill will contain one-twentieth of a grain. This forms a mass of an excellent consistence, and pills made therefrom retain their form and present an elegant appearance without the addition of any coating; they have but a faint odor of phosphorous, and that may be completely removed by the addition of oil of peppermint.

The experience gained from the administration of these pills in the in-and-out-patients' departments of the hospital to which I am attached proves that the therapeutic properties of the phosphorus are in no way injured or modified by this combination, but that it is fully equal to any that had been previously used.

In conclusion, I consider the advantages of this preparation to be that it is inoffensive to the tastes of the patient, definite and reliable for the prescriber, ready and convenient to the dispenser, and I believe, judging from its nature, it has unlimited keeping powers.—*Pharm. Jour.* (*London*), *Dec.* 6*th.*

